# Investigation of Immune Responses in Giant African Snail, *Achatina immaculata*, against a Two-Round Lipopolysaccharide Challenge

**DOI:** 10.3390/ijms241512191

**Published:** 2023-07-29

**Authors:** Xinfeng Wang, Yuzhe Tang, Zaiyuan Li, Qiang Wu, Xi Qiao, Fanghao Wan, Wanqiang Qian, Conghui Liu

**Affiliations:** 1 Shenzhen Branch, Guangdong Laboratory of Lingnan Modern Agriculture, Genome Analysis Laboratory of the Ministry of Agriculture and Rural Affairs, Agricultural Genomics Institute at Shenzhen, Chinese Academy of Agricultural Sciences, Shenzhen 518120, China; wangxinfeng0211@163.com (X.W.); tyz353107564@163.com (Y.T.); zaiyuanli01@163.com (Z.L.); wuqiang@caas.cn (Q.W.); qiaoxi@caas.cn (X.Q.); wanfanghao@caas.cn (F.W.); 2 School of Life Sciences, Henan University, Kaifeng 475004, China; 3 Shenzhen Research Institute, Henan University, Shenzhen 518000, China

**Keywords:** giant African snail, enhanced immune protection, transcriptome, hemocytes, lipopolysaccharide

## Abstract

As one of the 100 most-threatening invasive alien species, the giant African snail (*Achatina immaculata*) has successfully invaded and established itself in most areas of southern China. Protection against recurrent pathogen infections is vital to biological invasion. Enhanced immune protection has been previously found in other invertebrates, but not in the unique immune system of the giant African snail. In the present study, the survival rate of the giant African snail was recorded following a second infection with lethal doses of *Escherichia coli* after a previous first injection using lipopolysaccharide (LPS), and the mechanism of immune enhancement was investigated by examining the cellular and transcriptomic response of the giant African snail after two successive stimuli using LPS. Snails injected first with LPS, sterilized physiologic (0.9%) saline (SPS), phosphate-buffered saline (PBS) or untreated (Blank) were rechallenged at 7d with *E. coli* (Ec), and were named as LPS + Ec, SPS + Ec, PBS + Ec, Ec, and Blank. The log-rank test shows the survival rate of the LPS + Ec group as significantly higher than that of other control groups after the second injection (*p* < 0.05). By performing cell counting and BrdU labeling on newly generated circulating hemocytes, we found that the total hemocyte count (THC) and the ratio of BrdU-positive cells to total cells increased significantly after primary stimulation with LPS and that they further increased after the second challenge. Then, caspase-3 of apoptosis protease and two antioxidant enzyme activities (CAT and SOD) increased significantly after infection, and were significantly higher in the second response than they had been in the first round. Moreover, transcriptome analysis results showed that 84 differentially expressed genes (DEGs) were expressed at higher levels in both the resting and activating states after the second immune response compared to the levels observed after the first challenge. Among them, some DEGs, including Toll-like receptor 4 (TLR4) and its downstream signaling molecules, were verified using qRT-PCR and were consistent with the transcriptome assay results. Based on gene expression levels, we proposed that these genes related to the TLR signaling cascade participate in enhanced immune protection. All results provide evidence that enhanced immune protection exists in the giant African snail.

## 1. Introduction

A highly effective immune system is vital for protecting an organism from pathogenic infections and eliminating toxic or allergenic substances. The adaptive immune system, involving the production of antibodies or the generation of specific T-cell clones, produces immunological memory that allows for quick and effective responses upon a reencounter with the same pathogen [1]. In contrast, innate immune cells, such as granulocytes, macrophages, or natural killer (NK) cells, immediately and efficiently fight to kill a broad range of invading pathogens, but they do not provide specific or immunological memory for the purpose of host defense [2]. Because invertebrates do not carry the critical cells or molecules required for adaptive immunity, such as the T cells, B cells, and antibodies in vertebrates, it is generally believed that invertebrates lack canonical adaptive immunity [3]. However, the habitats of invertebrates are typically laden with infectious agents: viruses, bacteria, fungi, protists, and other animals. Many invertebrate groups have survived various infectious environmental pathogens, and it has been speculated that they have formed adaptability against the repeated infection of pathogens [4]. Previous research has shown that invertebrates show plasticity in their immune responses, which may form the basis for effective resistance to parasites and pathogens [5]. Recently, observations of invertebrates exposed to repeated infection have revealed that these animals exhibit immunological specificity and memory that is functionally equivalent to adaptive immunity in vertebrates [6,7,8].

Invertebrates can realize enhanced immune protection without the complexity of adaptive immunity, indicating that enhanced immune protection of invertebrates is much more complex than previously imagined [4]. Cellular immune responses include phagocytosis, cytotoxicity, aggregation and pathogen encapsulation, and the response to pathogen invasion is generally immediate [3]. Specifically, phagocytic hemocytes play a critical role in enhancing the immune protection of invertebrates [9]. For example, hemolymph phagocytosis of *Haliotis tuberculata* is enhanced after reinfection with *Vibrio harveyi* [10]. After a second challenge with *Vibrio splendidus*, the total hemocyte counts (THC) and number of regenerated hemocytes are markedly increased in oysters, and the enhanced phagocytosis by hemocytes is highly specific to *V. splendidus* [11]. After its initial exposure to *Eriocheir sinensis*, *Aeromonas hydrophila* was inactivated. However, it still showed an elevated phagocytosis rate of increased duration to such an extent as to increase survival against a second exposure to *A. hydrophila* [12]. In addition, several reports have demonstrated a range of receptors that, through interactions with ligands such as extracellular matrix proteins, hormones, growth factors, and cytokines, trigger specific immunocyte signaling cascades [3,13]. The immune response triggered via a sublethal dose of *Streptococcus pneumoniae*, protects *Drosophila melanogaster* against a subsequent lethal dose of *S. pneumoniae*. Achieving this protective effect requires phagocytes and Toll pathway activation [14]. In addition, researchers have found that Toll-like receptor (TLR) signaling molecules, together with antibacterial effectors, play indispensable roles in the enhanced immune protection of oysters [15]. These studies indicated the vital role that the enhanced immune protection of invertebrates plays in defending against recurrent infections by external pathogens.

The giant African snail *Achatina immaculata* is among the 100 most invasive species in the world and has gained a poor reputation as an agricultural pest [16]. Additionally, this species is an intermediate host for the nematode *Angiostrongylus cantonensis* responsible for eosinophilic meningitis [17]. They are currently considered among the most evolutionarily successful animals as, although they are without immunoglobulins, they have evolved distinct modalities, enabling them to detect and respond to microbial surface antigens [18]. Thus, their unique immune system and possibly enhanced immune protection may be factors that defend against *A. cantonensis* and promote the species’ high invasive potential, thus enabling them to cope with pathogens and the environmental stress of regions they have invaded. However, due to a lack of reference genomes, it has been difficult to investigate immune mechanisms at the overall omics level; therefore, the molecular mechanisms of enhanced immune protection remain unclear. In the present study, inspired by research into the enhanced immune protection of other mollusks and based on the high-quality reference genome obtained from our previous study, two different experiments were conducted [15,19]. In the first experiment, snails injected first with lipopolysaccharide (LPS) were rechallenged with *Escherichia coli* to detect the survival of the snail when infected with a lethal dose of *E. coli* after LPS infection. In the second experiment, two rounds of an immune elicitor, LPS, were injected to explore the enzymatic activity, hemocyte proliferation, and alterations in mRNA expression of immune-related genes during the first and second rounds of stimulation. The purposes were to determine whether enhanced immune protection exists in the giant African snail and understand the potential mechanism of enhanced immune protection.

## 2. Results

### 2.1. Survival Rate of Giant African Snail after Challenge with E. coli

**Figure 1:**
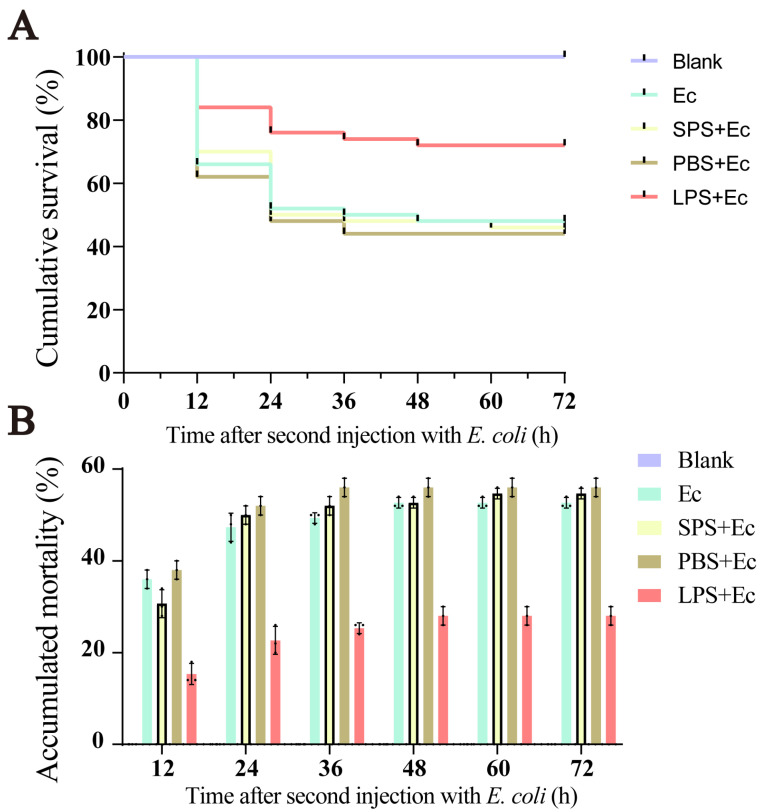
Kaplan-Meier survival curves for giant African snail after two consecutive infections. (**A**) Cumulative survival using the Kaplan–Meier method following the second injection with *E. coli* after the previous first injection with sterilized physiologic (0.9%) saline (SPS group), phosphate-buffered saline (PBS group), and lipopolysaccharide (LPS group) in giant African snail. (**B**) Accumulated mortality of different groups within 72 h after the second injection of *E. coli*. Blank group snails without any injections were employed as controls during two-round challenges. Ec group snails were without any treatment in first challenge.

We counted the number of the giant African snails that survived after two consecutive infections for the purpose of exploring the enhanced immune protection of the species. The results showed that no snails died in the Blank group (Figure 1). After the first injection, no mortality was observed in five groups. After the second infection, the log-rank test showed that the survival rate in the Ec group did not change significantly compared to that of the SPS + Ec and PBS + Ec groups (*p* > 0.05). But, the survival rate of the LPS + Ec group was significantly higher than other control groups after the second infection (*p* < 0.05).

### 2.2. Accelerated Regeneration of Circulating Hemocytes after the Second LPS Challenge

**Figure 2:**
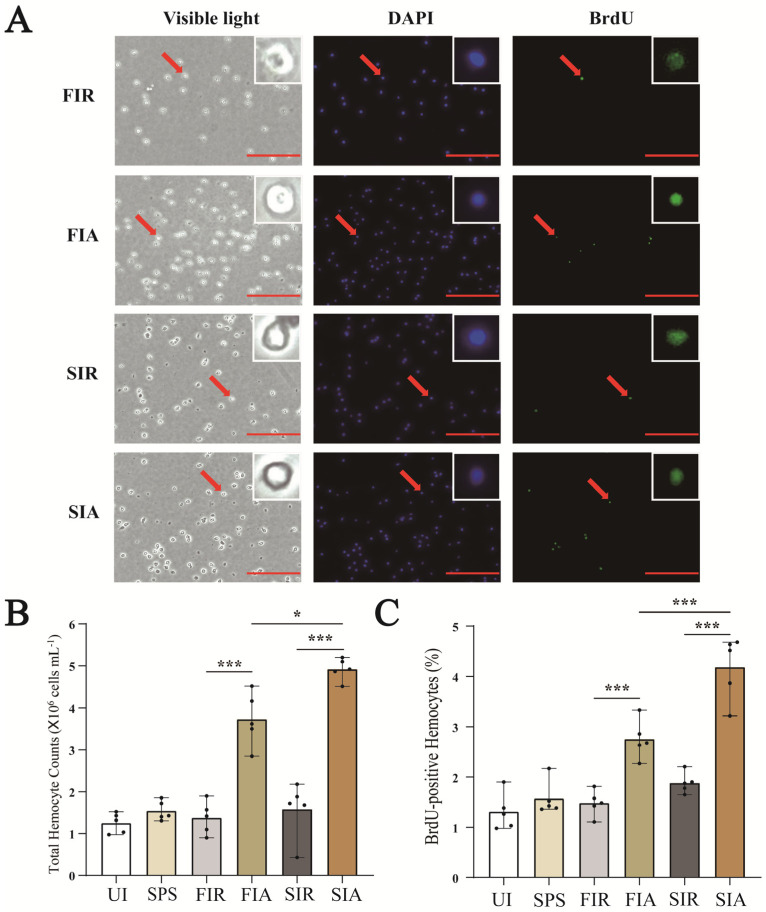
The number of hemocytes and newly generated circulating hemocytes of snails at 0 h (FIR group and SIR group) and 12 h (FIA group and SIA group) after both the first and second LPS injection. (**A**) Immunofluorescence staining of circulating hemocytes in the giant African snail after DAPI staining of nucleic acids (blue). The second antibody used for measuring the BrdU label was Daylight 488 (green). The red arrows indicate BrdU−positive hemocytes observed in the same area. Scale bar = 100 μm. The red arrows indicated the position of the enlarged cell image. (**B**) Effects of LPS on the THC of the giant African snail. (**C**) The significant differences in circulating BrdU−positive hemocytes after primary stimulation and the subsequent challenge with LPS (UI: snails without any treatment; SPS: snails received an injection of sterilized physiologic (0.9%) saline; FIR: immune−resting state of the first response; FIA: immune−activating state of the first response; SIR: immune−resting state of the second response; and SIA: immune−activating state of the second response; *: *p* < 0.05; ***: *p* < 0.01. The data demonstrated means of the five experiments (n = 5)).

The regenerated hemocytes were labeled with BrdU and immunostained with antibodies against BrdU (green) (Figure 2A). Compared with untreated snails, saline injection resulted in a slight increase in the number of hemocytes, but the results were not significant. In the first immune response, the number of hemocytes in the FIA group was significantly higher than that in the FIR group, and the ratio of the BrdU-positive cells to total cells was increased in the FIA group compared with the FIR group (*p* < 0.01) (Figure 2B,C). After the second challenge with LPS, the number of hemocytes was higher than that of all the other subgroups, and the ratio of newly generated circulating hemocytes also significantly increased in the SIA group compared with the SIR (*p* < 0.01) and FIA (*p* < 0.01) groups.

### 2.3. Apoptosis Protease and Antioxidant Enzymes Activity after First and Second Challenge with LPS

**Figure 3:**
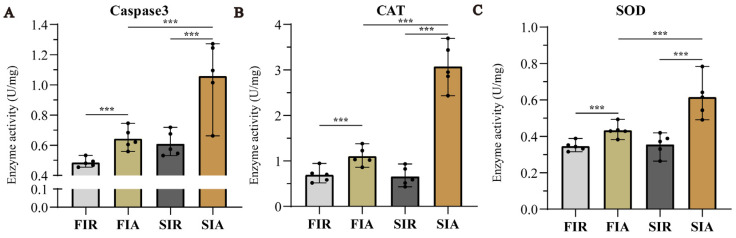
The apoptosis protease (caspase-3) activity and two antioxidant enzymes (SOD and CAT) activity assays. The snails hemocytes were sampled at 0 h (FIR group and SIR group) and 12 h (FIA group and SIA group) after both the first and second LPS injection. Then, enzyme activities of caspase−3, CAT, and SOD were determined using enzymatic activity assay kits. (**A**) Apoptosis protease enzymatic activity of the caspase-3. One unit of caspase3 is the amount of enzyme that will cleave 1.0 nmol of the colorimetric substrate Ac-DEVD-pNA per hour at 37 °C under saturated substrate concentrations. (**B**) Antioxidant enzymes activity of the CAT. One unit of CAT activity was defined as 1μmol H_2_O_2_ degraded per minute. (**C**) Antioxidant enzymes activity of the SOD. One unit of SOD activity was defined as the 50% decrease in superoxide anion reduction per minute. ***: *p* < 0.01. The data demonstrated means of the five experiments (n = 5)).

We performed two consecutive LPS infection experiments to investigate the relationship between immune protection and the LPS challenge. As an essential indicator of apoptosis, we detected caspase-3 activities. Although we did not use a positive control and activation blocker, the significant increase in caspase-3 in the giant African snail after LPS stimulation in this study was found to be like the reports on other mollusks [20,21]. The results showed that the activity of caspase-3 was obviously increased more in FIA and SIA than in FIR and SIR, respectively, and increased more in SIA than in FIA. In parallel, we also examined antioxidant enzymes activity (CAT and SOD) to measure antioxidant capacity (Figure 3). After the first LPS immune stimulation, the activity of two enzymes increased significantly. Then, two enzymatic activities decreased to the normal levels after 7 days. In the second immune responses, CAT and SOD activity were significantly increased, and higher levels were observed than in the first response.

### 2.4. Screening for Differentially Expressed Genes

Correlation analysis of gene expression levels among samples was performed to verify experimental reliability and sampling accuracy, and the sample correlations within groups were found to be higher than those between groups, indicating that the repeatability of experiments using the samples within each group was satisfactory. The overall expression level of the genes was estimated, and differences in coefficients of variation in gene expression levels among the four groups of samples were relatively small. The results of the differential gene expression analysis showed 7887 differentially expressed genes (DEGs) in the FIA group compared to the FIR (FIA/FIR) group, 5699 DEGs in SIR group compared to the FIR (SIR/FIR) group, 7259 DEGs in the SIA group compared to the SIR (SIA/SIR) group, and 8924 DEGs in the SIA group compared to the FIA (SIA/FIA) group.

### 2.5. The Genes Upregulated in Both the First and Second Immune Response

**Figure 4:**
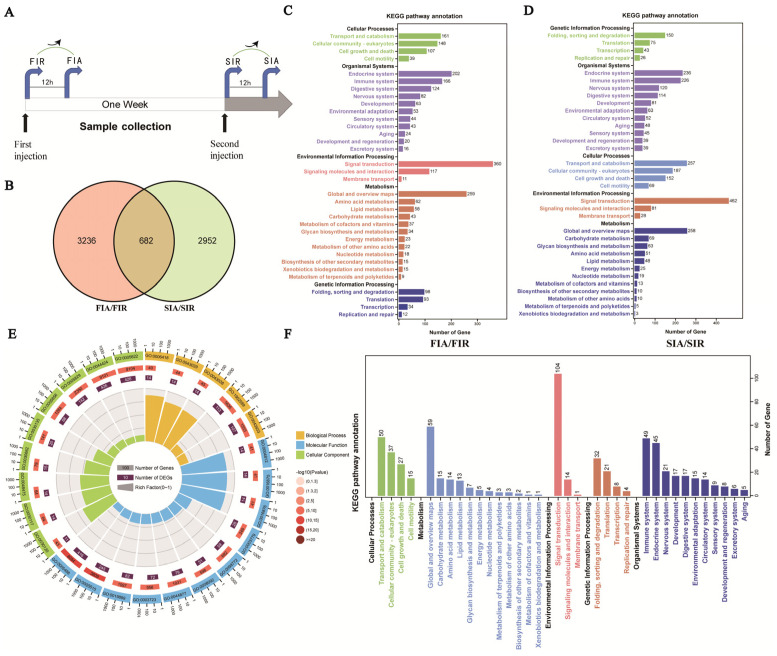
The results of genes up-regulated after either the first or the second LPS stimulations. (**A**,**B**) Screening for common or specifically upregulated genes during both the first and second LPS stimulation. (**C**) KEGG enrichment for the DEG (FIA vs. FIR). The up−regulated DEGs were mainly enriched in signal transduction of environmental information processing (360 DEGs involved), transport and catabolism (161 DEGs involved), folding, sorting and degradation (98 DEGs involved), and translation (96 DEGs involved) of genetic information processing, endocrine system (202 DEGs involved) and immune system (168 DEGs involved) of organismal system. (**D**) KEGG enrichment for the DEG (SIA vs. SIR). The up-regulated DEGs were mainly enriched in signal transduction of environmental information processing (462 DEGs involved), transport and catabolism (257 DEGs involved), folding, sorting and degradation (150 DEGs involved), and translation (75 DEGs involved) of genetic information processing, endocrine system (236 DEGs involved) and immune system (226 DEGs involved) of organismal system. (**E**) GO enrichment for the 682 common upregulated genes responsive to both the first and second LPS stimulations. The GO enrichment analysis was implemented by the one-tailed Fisher’s exact test with filter value of 0.001. (**F**) KEGG enrichment for the 682 common upregulated genes responsive to both the first and second LPS stimulations. The DEGs were mapped onto the KEGG database and enriched in each pathway of various categories. (Gene count represents the number of genes enriched in each term; FIR: immune−resting state of the first response; FIA: immune−activating state of the first response; SIR: immune−resting state of the second response; and SIA: immune−activating state of the second response).

A Venn diagram analysis was performed to identify candidate genes with specific and common upregulated DEG expression in the FIR/FIA group comparison (gene response to the first stimulation) and SIR/SIA group comparison (gene response to the second stimulation) (Figure 4A). A total of 6870 upregulated DEGs were identified, including 3236 DEGs specifically upregulated in the first immune response (FIA/FIR), 2952 DEGs specifically upregulated in the second immune response (SIA/SIR), and 682 unigenes upregulated in both immune responses (Figure 4B). Then, GO and KEGG pathway enrichments were performed on the DEGs to explore their potential biological functions. The results showed that the upregulated DEGs specific to the first stimulation were significantly enriched in terms of transport and catabolism (161), endocrine system (202), immune system (166), and signal transduction (360) (Figure 4C). In the second simulation, 257, 236, 226, and 462 genes were significantly enriched in KEGG terms in the second stimulation (Figure 4D). Furthermore, GO and KEGG enrichment analyses were performed to predict the function of 682 commonly upregulated DEGs. In the GO enrichment analysis, the upregulated DEGs were largely and significantly enriched in translation processes, such as tRNA aminoacylation for protein translation (GO:0006418), tRNA aminoacylation (GO:0043039), amino acid activation (GO:0043038), and aminoacyl-tRNA ligase activity (GO:0004812) (Figure 4E). The KEGG pathway analysis indicated that these upregulated DEGs were mainly enriched in terms of signal transduction (104), translation (21), and immune system capacity (49) (Figure 4F).

### 2.6. Genes Associated with Pre-Exposure and Enhanced Protective Effects

**Figure 5:**
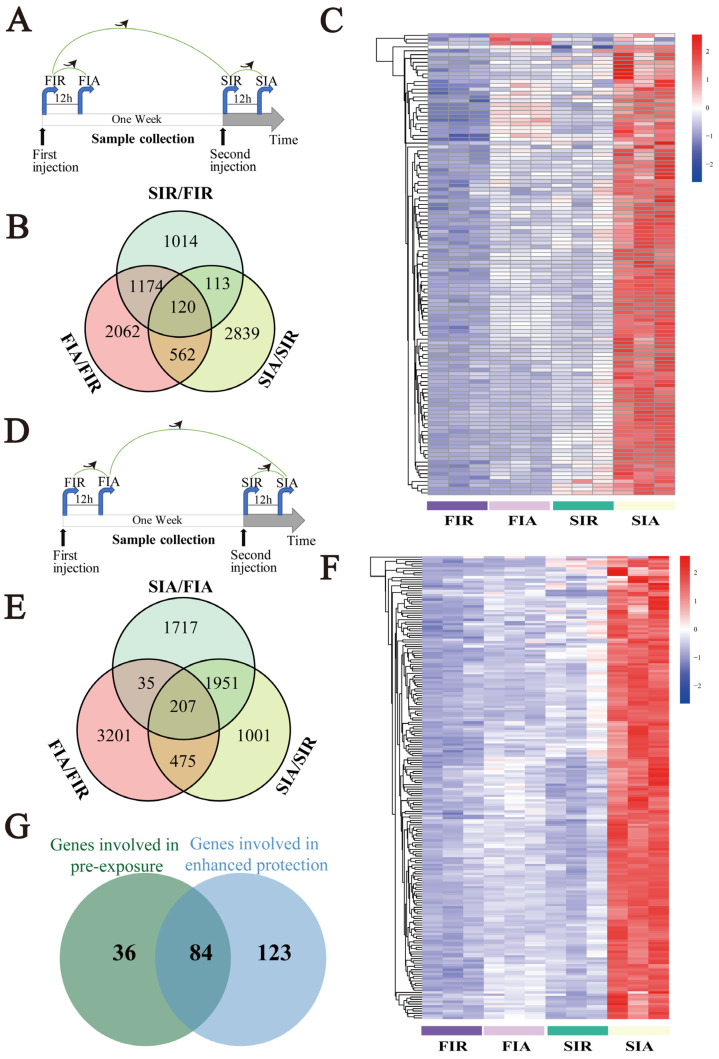
Screening of genes with higher expression levels at both resting and activating state in the second immune response than that in the first. (**A**,**B**) A total of 120 common genes were identified through comparisons of the FIA/FIR, SIA/SIR, and SIR/FIR groups. (**C**) The clustering expression profiles of 120 common genes expression levels in the four groups were shown by heat−maps that were drawn based on their FPKM value in each group. (**D**,**E**) A total of 207 common genes were identified through comparisons of the FIA/FIR, SIA/SIR, and SIR/FIR groups. (**F**) The clustering expression profiles of 207 common genes expression levels in the four groups were shown by heat−map, which was drawn based on their FPKM value in each group. (**G**) The identification of the 84 genes with higher express level at both resting (Venn B) and activating states (Venn E) in the second immune response than that in the first. (FIR: immune−resting state of the first response; FIA: immune−activating state of the first response; SIR: immune−resting state of the second response; and SIA: immune−activating state of the second response.).

To explore the pre-stimulation and the enhanced protective effects conferred by LPS, the expression of 682 commonly upregulated DEGs in hemocytes was further analyzed, and the results obtained for the resting (SIR/FIR) and activating (SIA/FIA) states were compared (Figure 5A,D). The results showed that 120 common DEGs were upregulated in the FIA/FIR, SIA/SIR, and SIR/FIR groups (Figure 5B). Among these DEGs, the heatmap showed a higher expression level for 120 DEGs in the immune-resting state of the second stimulation (the SIR group) than in the first response (the FIR group) (Figure 5C). In addition, 207 commonly upregulated DEGs were identified in the FIA/FIR, SIA/SIR, and SIA/FIA groups (Figure 5E). The heatmap showed a higher expression of 207 DEGs in the immune-activating state of the second stimulation (the SIA group) than in the first response (the FIA group) (Figure 5F).

To identify genes with lastingly high expression in hemocytes, common genes among the 120 identified upregulated DEGs in the immune-resting state and the 207 upregulated identified DEGs in the immune-activating state were subjected to a Venn diagram analysis (Figure 5G). The results revealed 84 common upregulated genes (Figure 5H), including DNA ligase 1, activating transcription factor 3 (ATF3), eukaryotic translation initiation factor 3 (eIF3), Kreppel-like factor, Toll-like receptor 4 (TLR4), ras-related protein Rac1, serine/threonine kinase Akt (AKT), a disintegrin and metalloproteinase 17 (ADAM17), and perforin 1 (PRF1). These genes were mainly involved in metabolic pathways, gene regulation, endocytosis, and immune pathways.

### 2.7. Validated Expression of Genes Associated with Pre-Exposure and Enhanced Protective Effects by qRT-PCR

**Figure 6:**
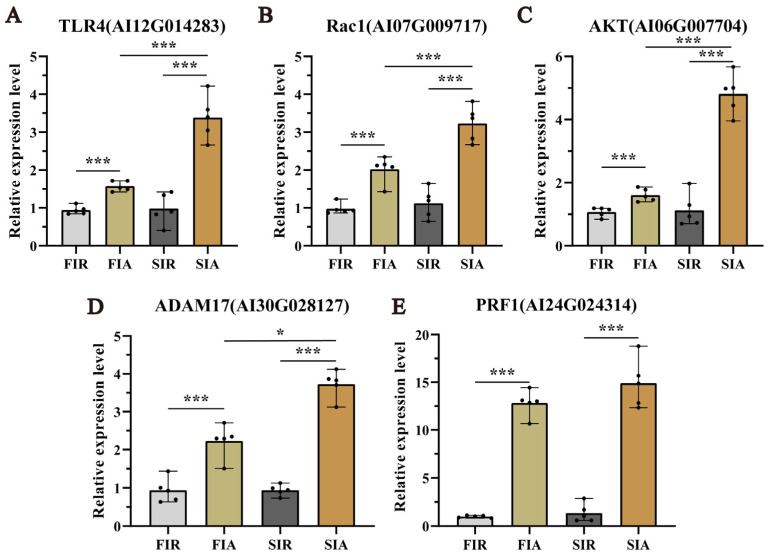
The expression profile of 5 selected genes mRNA in hemocyte post twice LPS challenge by real-time PCR. Snails hemocytes were sampled at 0 h (FIR group and SIR group) and 12 h (FIA group and SIA group) after both the first and second LPS injection. (**A**) The expression levels of TLR4. (**B**) The expression levels of Rac1. (**C**) The expression levels of AKT. (**D**) The expression levels of ADAM17. (**E**) The expression levels of PRF1. (FIR: immune−resting state of the first response; FIA: immune−activating state of the first response; SIR: immune−resting state of the second response; SIA: immune−activating state of the second response; *: *p* < 0.05; ***: *p* < 0.01. The data demonstrated means of the five experiments (n = 5)).

To evaluate the accuracy of the RNA-seq results, the expression of 5 genes, namely, TLR4, Rac1, AKT, PRF1, and ADAM17, was detected by qRT-PCR. As shown in Figure 6, the expression trends of the selected genes analyzed by qRT-PCR were consistent with the DEG analysis results. All the measured genes were significantly (*p* < 0.05) more highly expressed in the hemocytes of the FIA group than in those of the FIR (the first immune response) group and significantly (*p* < 0.05) more highly expressed in the hemocytes of the SIA group than in those of the SIR (the second immune response) group.

## 3. Discussion

An increasing number of studies have shown that the innate immune system of invertebrates exhibits memory in a fashion similar to that of the adaptive immune system operational in mammals. Further, this research has illustrated that this system endows the organism with increased resistance to reinfection after initial exposure to a pathogen [22,23]. Although enhanced immune protection has been observed, the detailed mechanisms at the molecular and cellular levels are not yet completely understood. With the completion of chromosome-level genomic analysis [19], we can obtain a glimpse into the possible molecular basis of enhanced immune protection in the giant African snail. In the present study, our results demonstrate that the first injection with LPS induced a higher expression of immune-related pathway genes in infected giant African snails, enhancing their immune protection against LPS re-exposure.

Enhanced immune protection is an important component of the immune defense against repeated pathogens of invading invertebrates with only innate immunity [24]. In the present study, the survival rate of giant African snails was recorded after a first injection with LPS and second challenge with *E. coli*. The results show that survival rate of the LPS + Ec group was significantly higher than that of other control groups after the second injection. Therefore, it was concluded that enhanced immune protection initiated after the primary exposure to LPS. In other invertebrates, the upregulation of the immune response after repeated infection is widespread, and it is considered to constitute a form of enhanced immune protection, such as *C. giga*, *H. diversicolo*, *B. glabrata*, and *A. japonicus* [10,15,21,25]. In addition, we performed a circulating hemocytes regeneration assay, an apoptosis protease (caspase-3) activity assay and two antioxidant enzyme (SOD and CAT) activity assays. The results demonstrated that total hemocyte counts (THC) increased significantly. Indeed, the apoptosis protease and antioxidant enzymes activity also significantly increased when giant African snails encountered the secondary LPS challenge. Firstly, LPS exposure increased the THC and the ratio of newly generated circulating hemocytes, and these significantly increased after the second challenge compared with after the first challenge. Circulating hemocytes are essential in the immune response against infection in invertebrates [26], interacting with numerous foreign particles in a way that leads to the subsequent activation of cellular immune reactions [3]. Therefore, the increased number of hemocytes potentially represents stronger and faster immune responses. In other studies, oyster re-exposure to *V. splendidus* has been shown to cause a marked increase in the total hemocyte count (THC), and the phagocytic rate was also enhanced significantly [11]. Secondly, the contrast in the apoptosis protease (caspase-3) and antioxidant enzymes (CAT and SOD) activity between the FIA and SIA groups indicates that the immune protection of LPS was enhanced when they were infected again. Caspase-3 is the primary executioner caspase in apoptosis [27]. Apoptosis has been shown to play a vital role in many biological processes and is responsible for destroying redundant, dysfunctional, or damaged cells [28]. Hemocytes had a high level of caspase-3 activity, indicating that increased immune capacity played a role in the elimination of foreign pathogens. For instance, in oysters, caspase-3 in hemocytes increased significantly after LPS stimulation, and this suggested that caspase-3 was able to bind diverse PAMPs and activate the response against pathogen invasion [21]. Meanwhile, SOD and CAT are key antioxidant enzymes involved in protection against immune infection and oxidative stress. Antioxidant enzymes directly scavenge free radicals and related reactants, and is the first defense line in organisms [29]. These enzymatic activities were markedly elevated, suggesting raised body antioxidation against infections. In *S. paramamosain*, the CAT and SOD activity of hemocytes were significantly increased, leading to enhanced protection of the host against microbial infections [30]. The significant changes in these enzyme activities against the two-round challenge of LPS demonstrate that enhanced immune protection exists in the giant African snail at the enzyme level. Thus, it was inferred that the primary stimulation of LPS improved apoptosis and the antioxidant effect by increasing the level of enzymatic activities and played a priming role in this process, contributing to a burst in the immune response production during the second exposure to LPS. Based on these experimental results, we observed that an infection-enhanced giant African snail immunity to secondary infection with the same pathogen. The observation of gene expression also validated the findings. In transcriptome data, significantly more upregulated DEGs were enriched in the KEGG pathway “immune system” after the second stimulation (226/3634, immune system/total DEGs) than after the first stimulation (166/3910) (Fisher’s exact test, *p* < 0.01). This result revealed that the transcript levels in the second immune response were more efficient than those in the first response. In oysters, a similar result has also been reported, with an increase in the already more concentrated gene expression after exposure to *V. splendidus* [15]. Overall, we think that enhanced immune protection is present in the giant African snail. Additionally, we observed that primary stimulation with LPS may have triggered stronger immune responses after the second challenge with LPS.

Pathogen-associated molecular patterns (PAMPs) can be recognized by pattern recognition receptors (PRRs) in order to trigger downstream signal transduction pathways and lead to a rapid immune response capable of eliminating invading microorganisms [31]. LPS, a critical structural component of the outer membrane of Gram-negative bacteria, is considered a classical PAMP in exploring the regulation of the immune system [32,33]. For instance, LPS has been used to investigate the molecular basis of the immune response to microbial diseases in *Ruditapes philippinarum* [34]. In this study, we employed LPS as an immune elicitor instead of a Gram-negative pathogen. Gene expression changes in the giant African snail post-LPS stimulation may represent common characteristics in Gram-negative bacterial infection and can be used to explore the dynamic signaling pathways in an immune-balanced state. LPS from different Gram-negative bacteria share many structural and functional commonalities [35]. However, in contrast to pathogens, the signaling cascades triggered by LPS do not interfere with host homeostasis and do not lead to hypersensitivity or silencing of the immune response [36]. Moreover, PAMPs have also been used to identify and characterize PRR repertoires in invertebrates [37]. We found that multiple PRRs were activated by LPS stimulation. For example, CTLs, galectins, TEPs, FREPs, and TLRs were upregulated after the first stimulation, and the second response evoked a higher degree of TLR expression. In oysters, TLR6 has been shown to display high levels of expression for a long time after being subjected to two *V. splendidus* stimulations [15]. On the basis of these results, we speculated that the recognition of LPS by these PRRs may cause a priming effect. Taken together, these results suggest that LPS-triggered enhanced immune protection at the cellular, enzyme activity, and transcriptome levels in giant African snails through pattern recognition may be a general immune defense strategy against Gram-negative bacteria.

TLRs, one of the major classes of PRRs, are known to play essential roles in innate immune defense against invading pathogens [29]. TLR4 is among the most characterized TLRs that have been identified to sense bacterial invasion and subsequent involvement in the immune response of mollusks [38]. Here, several upregulated genes, related to the TLR signaling cascade, were identified, including TLR4, Rac1, and AKT (also known as protein kinase B). Some studies have found that LPS stimulated TLR4 in mollusks [39], resulting in Rac1 and AKT activation [40,41]. Simultaneously, the expression levels of downstream effectors in the TLR4 signaling were measured, and the expression of ADAM17 and PRF1 was significantly upregulated after the second stimulation. As reported, NF-κB released by AKT activates IκB kinase (IKK) and can bind directly to the ADAM17 and PRF1 promoters to enhance their expression [42,43,44]. Furthermore, ADAM17 can trigger the release cytokines and other inflammatory proteins in a way that eliminate foreign antigens, and PRF1 can directly target Gram-negative bacteria and certain pathogenic parasites by forming pores on the membranes of target cells, eventually causing cell lysis [43,44,45,46]. In summary, the TLR4 signaling cascades is speculated to participate in enhanced immune protection, and its possible mechanism of action is mediated through TLR4 that has first been activated by LPS exposure, with the subsequent activation of the signaling cascade promoting downstream antibacterial effector production.

## 4. Materials and Methods

### 4.1. The Giant African Snail and Microbes

Adult snails were collected from a local farm in Shenzhen, Guangdong Province, China, and acclimated for a week under laboratory conditions (25 ± 2 °C) before use in experiments. The average weight of these adult snails was approximately 70 g, and the average size of the adults was approximately 6 cm in length and 4 cm in width. The *Escherichia coli* strains were cultivated in Luria-Bertani (LB) medium at 37 °C, and harvested by centrifugation at 7000× *g* for 5 min. The pellet was washed and resuspended in sterilized physiologic (0.9%) saline (SPS), and the suspension was adjusted to a final concentration of 107 CFU/mL termed the “*E. coli* suspension”. LPS from *E. coli* 0111:B4 (Sigma-Aldrich, St. Louis, MO, USA) was dissolved in phosphate-buffered saline (PBS; 0.14 M NaCl, 3 mM KCl, 8 mM NaH_2_PO_4_.12H_2_O, and 1.5 mM KH_2_PO_4_, pH 7.4) at a concentration of 0.5 mg ml^−1^.

### 4.2. Survival Rate after Bacterial Challenge

The immune stimulation schedule was based on that of a previous study [15,19,47]. The experiment was split into the first response and second response. Thirty snails were maintained in laboratory conditions without any treatment and were referred to as Blank-group snails. For the first injection, the experimental groups were designed as an SPS group (100 snails received an injection of 100 μL sterilized physiologic (0.9%) saline), a PBS group (100 snails received an injection of 100 μL phosphate-buffered saline) and an LPS group (100 snails received an injection of 100 μL LPS). After 7d, in the second injection, these groups of snails received an injection of *E. coli* suspension with the effective CFU per gram of snails of 5.0 × 10^4^. The groups of LPS, PBS, SPS, and untreated in first injection were named LPS + EC, PBS + EC, SPS + EC, and EC, respectively, after the second injection of *E. coli*. The concentrations of *E. coli* mentioned above were determined by the mortality of snails after the injection of *E. coli* in the pre-experimental stage. The survival rate of snails was measured in different groups after the challenge with *E. coli*. A total of 100 snails were used in each group and survival was followed for 3 days after infection with *E. coli*. This survival experiment was repeated three times. Survival rates were calculated with log-rank test on Kaplan–Meier survival curves in GraphPad Prism to assess statistical differences between treatments.

### 4.3. Hemocyte Collection

**Figure 7:**
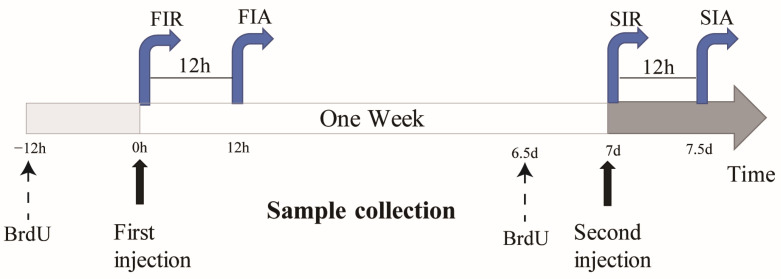
Schematic diagram showing the experimental design. For the first exposure of snails, a first pre-injection of 100 μL of LPS was conducted. One week later, a second injection of live 100 μL of LPS was performed. The snails hemocytes were sampled at 0 h (FIR group and SIR group) and 12 h (FIA group and SIA group) after both the first and second LPS injection. Then, enzymes activity assay and cell counting were performed and gene expression levels of hemocytes were assessed. (FIR: immune-resting state of the first response; FIA: immune-activating state of the first response; SIR: immune-resting state of the second response; SIA: immune-activating state of the second response; and BrdU: Bromodeoxyuridine; dotted line represent use for a partial experiment).

In order to explore the immune responses in giant African snails against the two-round challenge, LPS was used to induce first and second immune responses in the giant African snails, and PBS was used as the control (Figure 7). To elicit the first immune response, 100 μL of LPS was injected into snails, and the snails were sampled 0 and 12 h after stimulation, which were considered to reflect the immune-resting state of the first response (FIR) and immune-activating state of the first response (FIA), respectively. The second stimulation was elicited in the same manner after seven days, and the snails were allocated into an immune-resting state of the second response (SIR) and an immune-activating state of the second response (SIA) group. For hemocyte collection, hemolymphs were pooled together as one biological repeat from each group of 5 snails and were immediately centrifuged at 800× *g* and 4 °C for 10 min in order to harvest hemocytes.

### 4.4. Bromodeoxyuridine (BrdU) Incorporation Assay

BrdU (Yeasen, 50 mM) in a 100 μL solution was injected into the snail muscle 12 h prior to collection. Then, 10 mL hemocyte suspensions (1:1 volume ratio of hemolymph to anticoagulant) in each biological repeat (taken at 4 time-points for 4 groups in 5 replicates) were used to perform a hemocyte regeneration analysis based on a method described in a previous report [11,48]. The hemocytes in each sample were deposited on a clean glass slide treated with 4% paraformaldehyde and allowed to adhere for 1 h in a wet chamber at room temperature. After the hemocytes formed monolayers on the slides, the slides were washed three times with PBS. The slides with these hemocytes were prepared as previously described, and BrdU assays were performed using a BrdU cell proliferation assay kit (Servicebio, Wuhan, China) following the manufacturer’s protocol. Briefly, the fixed slides were incubated with 1 M HCl at 37 °C for 30 min, followed by rinsing with PBS. Subsequently, the slides were preincubated with 3% bovine serum albumin (BSA) at 37 °C for 30 min and then incubated with anti-BrdU mouse mAb (1:1000 dilution in 1% BSA) at 37 °C for another 3 h. After rinsing with PBS, the slides were incubated with Daylight 488-coupled goat anti-mouse IgG (1:1000 dilution in 1% BSA, Wuhan, China) at 37 °C for 1 h and counterstained with 1 mL of DAPI (Servicebio, Wuhan, China). The cells were counted under a fluorescence microscope, and the ratio of BrdU-positive cells to total cells was calculated.

### 4.5. Determination of Apoptosis Protease and Antioxidant Enzymes Activity

Enzyme activities of caspase-3, catalase (CAT), and superoxide dismutase (SOD) were determined using enzymatic activity assay kits (caspase-3: Beyotime, Shanghai, China; CAT: Boxbio, Beijing, China; SOD: Solarbio, Beijing, China). Briefly, these samples were collected as described above, i.e., we performed the collection of 0.5 mL of serum in serum collection tubes and transferred them to a new 96-well plate. The caspase-3, CAT, and SOD activity were analyzed by the assay kit according to the manufacturer’s protocol and previous research [49,50,51], and the results were then detected at 405 nm, 240 nm, and 560 nm by a microplate reader, respectively. One unit (U) of caspase-3 is the amount of enzyme that will cleave 1.0 nmol of the colorimetric substrate Ac-DEVD-pNA per hour at 37 °C under saturated substrate concentrations. One unit of CAT activity was defined as 1 μmol H_2_O_2_ degraded per minute. One unit of SOD activity was defined as the 50% decrease in superoxide anion reduction. The enzyme activity was expressed as units per mg protein (U/mg protein).

### 4.6. Library Preparation and Sequencing

Total RNA was extracted from hemocytes (5 × 106 cells) using a TRIzol reagent (Invitrogen) in a method performed according to the manufacturer’s instructions. The extracted RNA was quantified with a Nanodrop (2000) (Thermo Scientific) and checked for integrity with an Agilent 2100 Bioanalyzer (Agilent Technologies). Then, mRNA was pulled down by beads with poly-T to construct cDNA libraries (inserting 350 bp) using a VAHTS TM mRNA-seq V3 Library Prep Kit (NR611) and sequenced on an Illumina HiSeq 2500 sequencer.

### 4.7. Bioinformatics

The giant African snail genome sequence and annotation files [19] are available at NCBI (https://www.ncbi.nlm.nih.gov/ (accessed on 22 December 2020)). Raw read was processed into clean reads by trimming the adapter sequence and low-quality reads. Then, reads obtained by RNA-sequencing (RNA-seq) were mapped to the reference giant African snail genome using TopHat2 (version 2.1.0) [52] with default settings. The level of mRNA expression was evaluated using StringTie (version 2.1.1) [53] by calculating FPKM (fragments per kilobase of transcript per million mapped reads). Differentially expressed genes (DEGs) were identified between the two groups using the DESeq2 R package [54], and we considered adjusted-P (Padj) ≤ 0.05 and |log2FoldChange| ≥ 1 as significant. GO functional enrichment and KEGG pathway enrichment analyses were performed using the GO database (http://geneontology.org (accessed on 23 December 2020)) and KEGG database (http://www.genome.jp/kegg/ (accessed on 23 December 2020)), respectively, and the results were visualized using the ggplot2 package.

### 4.8. Gene Expression Validation

Quantitative real-time PCR was performed to validate the expression levels of 5 selected genes, namely, TLR4 (AI12G014283), Rac1 (AI07G009717), AKT (AI06G007704), PRF1 (AI24G024314), and ADAM17 (AI30G028127). Two independent real-time PCRs were performed with cDNA from each biological replicate. In order to compare the relative expression of these 5 genes in the samples, the housekeeping gene EF1α (AI13G015621) was used as the internal control. RNA preparation was carried out as described above. First-strand cDNA was synthesized from approximately 1 μg of total RNA using a Hifair^®^ III 1st Strand cDNA Synthesis Kit (Yeasen, Shanghai, China) in a 20-μL reaction system following the manufacturer’s protocol. A SYBR Green real-time PCR assay was carried out in an ABI PRISM 7500 Sequence Detection System (Applied Biosystems) according to the manufacturer’s instructions. The thermal cycling parameters were 95 °C for 30 s, followed by 40 cycles of 95 °C for 10 s and 60 °C for 30 s. Experiments were performed in five biological replicates and two technical replicates, and the data were calculated using the 2−DDCT method [55].

### 4.9. Statistics Analysis

Statistical analyses were carried out by using SPSS 13.0 statistical software. The statistical analysis of the hemocyte count and enzyme activity was calculated using two-way ANOVA. The data were tested by SPSS software for their normal distribution and equal variance before ANOVA analysis. The statistical analysis of gene relative expression used the comparative CT method (∆CT = CT of target gene − CT of EF1α and ∆∆CT = ∆CT of any sample—the calibrator sample). The results of immune gene expression change in the first stimulation (FIA_FIR) and second stimulation (SIA_SIR) used Fisher’s exact test. Differences were considered significant at *p* < 0.05.

## 5. Conclusions

In the present study, the higher survival rate in the LPS + Ec group with the first injection of LPS and second injection of live *E. coli* provided evidence of enhanced immune protection in the giant African snail. We also described the general immune response characteristics of enhanced immune protection caused by Gram-negative bacteria. First, the primary stimulation with LPS induced hemocyte proliferation and activated apoptosis protease and antioxidant enzymes, significantly enhancing immunity against the second challenge. Second, the TLR4 signaling cascade was activated to participate in the enhanced immune protection of cellular, enzyme activity, and transcriptome in the giant African snail, possibly by modulating TLR4 and its downstream signaling molecules to produce downstream effector molecules that eliminate foreign pathogens after reinfection. This study provides further insight into cellular immune responses, antioxidant enzyme activity, and transcriptomic expression in the giant African snail and contributes to a greater understanding of the mechanism of enhanced immune protection in invertebrates.

## Data Availability

The transcriptome data has been submitted to NCBI with the SRA accession number SRR22141245-SRR22141256.
